# Role of Toll-Like Receptor 4 on Lupus Lung Injury and Atherosclerosis in LPS-Challenge ApoE^−/−^ Mice

**DOI:** 10.1155/2013/476856

**Published:** 2013-09-15

**Authors:** Jing-qin Ni, Qiufang Ouyang, Ling Lin, Ziyang Huang, Huixia Lu, Xiaoqing Chen, Huili Lin, Zhenhua Wang, Dongming Xu, Yun Zhang

**Affiliations:** ^1^Cardiovascular Department, Second Affiliated Hospital and Second Clinical Medical College, Fujian Medical University, Quanzhou, Fujian 362000, China; ^2^Rheumatism Department, Second Affiliated Hospital and Second Clinical Medical College, Fujian Medical University, Quanzhou, Fujian 362000, China; ^3^Cardiology Department, Second Affiliated Hospital of Fujian Medical University Zhongshan North Road 34, Quanzhou, Fujian 362000, China; ^4^Key Laboratory of Cardiovascular Remodeling and Function Research, Chinese Ministry of Education and Chinese Ministry of Health, Shandong University Qilu Hospital, Jinan, Shandong 250012, China

## Abstract

To investigate the pathologic mechanisms of toll-like receptor 4 (TLR4) in lung injury and atherosclerosis, ApoE^−/−^ or wild-type mice were intraperitoneally administered saline, lipopolysaccharides (LPS), or LPS plus TAK-242 (TLR4 inhibitor), respectively, twice a week for 4 weeks. Serum autoantibody of antinuclear antibody (ANA), anti-double-stranded DNA (anti-dsDNA), and cytokines of interferon-gamma (IFN-**γ**), tumor necrosis factor (TNF-**α**), and interleukin-1 (IL-1**β**) were assessed by ELISA. Hematoxylin and eosin (HE) and Perl's stains for lung pathomorphology as well as HE staining for atherosclerosis were employed. TLR4 in macrophages was detected by double immunofluorescent staining. While protein expressions of TLR4, nuclear factor-kappa B p65 (NF-**κ**B p65), and B cell activating factor belonging to the TNF family (BAFF) were examined by immunohistochemistry. We found that serum autoantibody (ANA and anti-dsDNA), cytokines (IFN-**γ**, TNF-**α**, IL-1**β**), lung inflammation, and intima-media thickness in brachiocephalic artery were obviously increased after LPS challenge in both genotypes, but to a lesser extent in wild-type strains. And those alterations were alleviated by coadministration of LPS and TAK-242. Mechanistically, upregulation of TLR4, NF-**κ**b, and BAFF was involved. We concluded that TLR4/NF-**κ**b/BAFF in macrophages might be a possible common autoimmune pathway that caused lung injury and atherosclerosis. TLR4 signal will be a therapeutic target in atherosclerosis and immune-mediated lung injury.

## 1. Introduction

Systemic lupus erythematosus (SLE) is multiorgan system autoimmune disease characterized by frequent cardiovascular diseases and fatal alveolar hemorrhage (AH) comorbidities [1]. Recently, studies on the precise mechanisms underlying these comorbid disorders are springing up. Bessant et al. [2] reported a complex interplay of traditional and lupus-specific risk factors accelerated atherosclerosis (AS) development in SLE. Meanwhile, Barker et al. [3] indicated the pathogenesis of AH was implicated in B cells as evidenced by reduced AH prevalence in Rag1^−/−^ or Igmu^−/−^ mice following pristane injection. However, the divergent findings on this comorbidity of autoimmune disorder, atherosclerosis, and lung inflammation highlight the considerable heterogeneity.

Indeed, there is further evidence to support the hypothesis that TLR4 signaling is relevant to atherosclerosis and lupus lung injury [4, 5]. Lee et al. [6] demonstrated the activation of immature antigen presenting cells through TLR4 signaling correlated with increased serum interferon-gamma (IFN-*γ*), interleukin-10 (IL-10), and anti-double-stranded deoxyribonucleic acid (anti-dsDNA) production in anti-dsDNA transgenic mice. Similarly, overexpression of TLR4 in mice has led to the production of anti-dsDNA and the immune complex-mediated glomerulonephritis, suggesting that TLR4 signaling plays critical roles in lupus progression [7]. Furthermore, in ApoE^−/−^ mice following lipopolysaccharides (LPS) injection, atherosclerotic lesion size was increased, whereas it was reduced when there is deficiency in TLR4 [8, 9]. Additionally, our previous study [10] verified that LPS can successfully induce lupus-like symptoms in ApoE^−/−^ mice characterized by high titers of antinuclear antibodies (ANA) and anti-dsDNA antibodies. These collective observations raised the possibility that TLR4 signaling might serve as a bridge between lung damages and atherosclerotic lesions in SLE. However, to our knowledge, no study has attempted to elaborate the role of the TLR4 signal on the interrelations of these disorders as yet.

Monocytes and macrophages are an essential arm of the innate immune system with a multitude of immunological functions, including antigen presentation, phagocytosis, and cytokine production. Emerging evidence showed aberrations of monocyte/macrophage phenotype and function in AS. However, the data on the role of macrophages on SLE complicated with AS and hemorrhagic pulmonary capillaritis are scarce. The purpose of this research was to investigate the pathologic mechanisms of TLR4 in lung injury and AS after LPS was administered to ApoE^−/−^ mice.

## 2. Materials and Methods

### 2.1. Mice and Materials

All of the procedures and protocols were approved by *Animal Care Committee of Shandong University* and followed the guidelines of *Animal Management Rules of the Chinese Ministry of Health* (Document No. 55, 2001). Thirty ApoE^−/−^ and thirty wild-type mice on C57BL/6 background (female, 10 weeks old) were obtained from Peking University Animal Center (Beijing, China). Mice were fed on a high-fat diet containing 0.25% cholesterol and 15% cocoa butter under standardized lighting conditions (12 h light-dark cycle) and temperature (21 ± 1°C). And mineral water was administered ad libitum. Mice of both genotypes were randomly assigned to LPS or LPS + TAK-242 or saline administration. LPS (2.5 mg/kg), LPS (2.5 mg/kg) plus TAK-242 (0.3 mg/kg) and saline were administered respectively by intraperitoneal injection, twice a week for 4 weeks. At the end of experiments, all mice underwent euthanasia with injection of overdose pentobarbital (50 mg/kg). LPS from *Escherichia coli* 055 : B5 was purchased from Sigma Chemical. And TAK-242, (TLR4 inhibitor) was from InvivoGen.

### 2.2. Enzyme-Linked Immunosorbnent Assay (ELISA) for Autoantibody and Cytokines in Serum

Sera autoantibody of ANA (Alpha Diagnostic International), anti-dsDNA (Alpha Diagnostic International), and cytokines of IFN-*γ* (Dakewe biotech), tumor necrosis factor (TNF-*α*, Dakewe biotech), and IL-1*β* (Dakewe biotech) were analyzed with ELISA kits according to the instructions. All measurements were carried out in duplicate. 

### 2.3. Hematoxylin and Eosin (HE) and Perl's Stains for Lung Injury as well as HE Staining for Atherosclerosis Assessment

The lung tissue sections were stained with hematoxylin and eosin (HE). Then Perl's staining was performed for ferric iron assessment. For Perl's staining, lung slides were incubated for 10 min in a stain containing hydrochloric acid and potassium ferricyanide and then counterstained with eosin and Mayer's haemalum.

Serial transverse cryosections of 5 *μ*m in thickness were obtained from the brachiocephalic arteries and aortic sinus and were selectively stained with HE at 50 *μ*m intervals.

### 2.4. Double Immunofluorescent Staining for TLR4 Expression in Macrophages

For localization of TLR4 expression, TLR4 (1 : 50; Novus) and anti-F4/80 (1 : 100; Abcam) were used by immunofluorescence staining for tissue macrophages in lung sections and plaques in aortic sinus.

### 2.5. Immunohistochemistry for NF-*κ*B p65, BAFF Expression in Lung and Aortic Sinus

Immunohistochemistry was performed. Aortic sinus frozen sections and lung paraffin sections were employed to determine nuclear factor-kappa B p65 (NF-*κ*B p65, 1 : 150; Abcam) and B cell activating factor belonging to the TNF family (BAFF, 1 : 100; Abcam) expressions. The immunostained areas were analyzed with Image Pro-Plus 6. Ten fields were chosen randomly for each specimen.

### 2.6. Statistical Analysis

All values are expressed as the mean ± SD unless otherwise indicated. Comparisons between groups were tested by one-way ANOVA, followed by LSD test in the condition of variance homogeneity or Tamhane test when variances are unequal. All reported probability values are 2-tailed, and *P* value of 0.05 was accepted for significance. Statistical analyses were performed using SPSS 17.0.

## 3. Results

### 3.1. Autoantibody in LPS-Challenged ApoE Knockout and Wild-Type Mice

As detailed in Table 1, the serum ANA and anti-dsDNA in ApoE^−/−^ mice were significant higher than those in wild-type mice after exposure to saline. ApoE^−/−^ mice showed a markedly increase in ANA and anti-dsDNA, while wild-type mice revealed a smaller but still significant increase after LPS stimulation. Furthermore, TAK-242 could apparently reduce the serum anti-dsDNA levels in both genotype mice. However, it diminished ANA marginally in both strains mice.

### 3.2. Levels of Inflammatory Cytokines IFN-*γ*, TNF-*α*, and IL-1*β* in Sera

**Figure 1:**
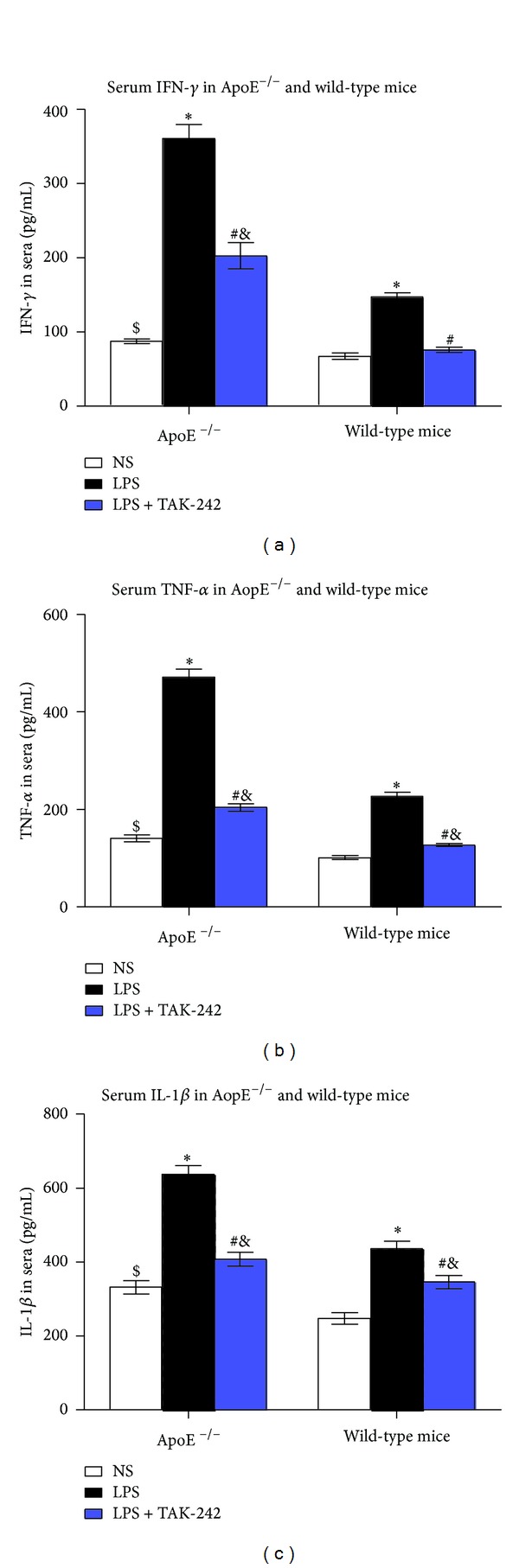
Levels of inflammatory cytokines IFN-*γ*, TNF-*α*, and IL-1*β* in sera (pg/mL). ^∗&^ *P* < 0.01 versus corresponding NS group, ^#^ *P* < 0.05 versus corresponding LPS group, and ^$^ *P* < 0.05 versus wild-type mice. IFN-*γ*: interferon gamma; TNF-*α*: tumor necrosis factor; IL-1*β*: interleukin-1*β*; NS: physiological saline; LPS: lipopolysaccharide; TAK-242: resatorvid.

As compared with their corresponding NS control, the levels of IFN-*γ* and TNF-*α* were dramatically increased in ApoE^−/−^ mice and wild-type mice, and to a lesser extent in wild-type strains after LPS intervention (Figure 1). LPS-induced IFN-*γ*, TNF-*α*, and IL-1*β* production was increased by 3.1-fold, 2.3-fold, and 0.9-fold, respectively, in ApoE^−/−^ mice while by 1.2-fold, 1.3-fold, and 0.7-fold in wild-type mice. Alternatively, IFN-*γ*, TNF-*α*, and IL-1*β* production was markedly inhibited by TAK-242, but their concentrations were still greatly higher than those in NS-treated counterparts.

### 3.3. Aggravated Haemorrhage Pulmonary Capillaritis in LPS-Primed ApoE^−/−^ Mice

**Figure 2:**
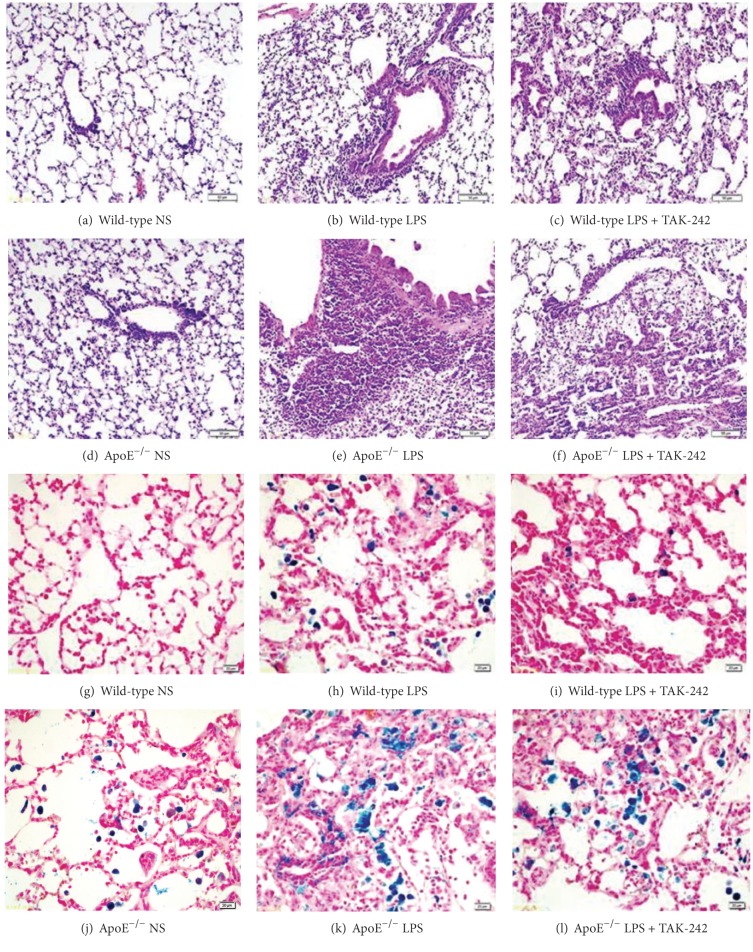
Haemorrhage pulmonary capillaritis in lung of the ApoE^−/−^ and wild-type mice. Sporadic hemosiderin-laden macrophages were highlighted by Perl's staining for iron (stained blue). Enhanced haemorrhage pulmonary capillaritis was found in LPS-primed ApoE^−/−^ mice by HE ((a)–(f), 200x) and Perl's staining ((g)–(l), 400x).

In both mouse strains primed by LPS, inflammatory infiltration of lymphocytes, plasma cells, and polymorphonuclear leucocytes could be observed around the capillaries and venules as well as hemosiderin-laden macrophages in alveolar spaces and interstitium. While those pathological changes were more pronounced in ApoE^−/−^ mice than in wild-type counterparts, as depicted by HE and Perl's staining (Figure 2). The prevalence of alveolar hemorrhage in LPS-exposed ApoE^−/−^ mice was significantly higher (70%, *n* = 10) versus wild-type mice (40%, *n* = 10). And the incidence was 30% in NS-treated ApoE^−/−^ mice. TAK-242 could partly improve the symptoms of lung inflammation and hemorrhage. 

### 3.4. Accelerated Atherosclerosis after LPS Intervention

**Figure 3:**
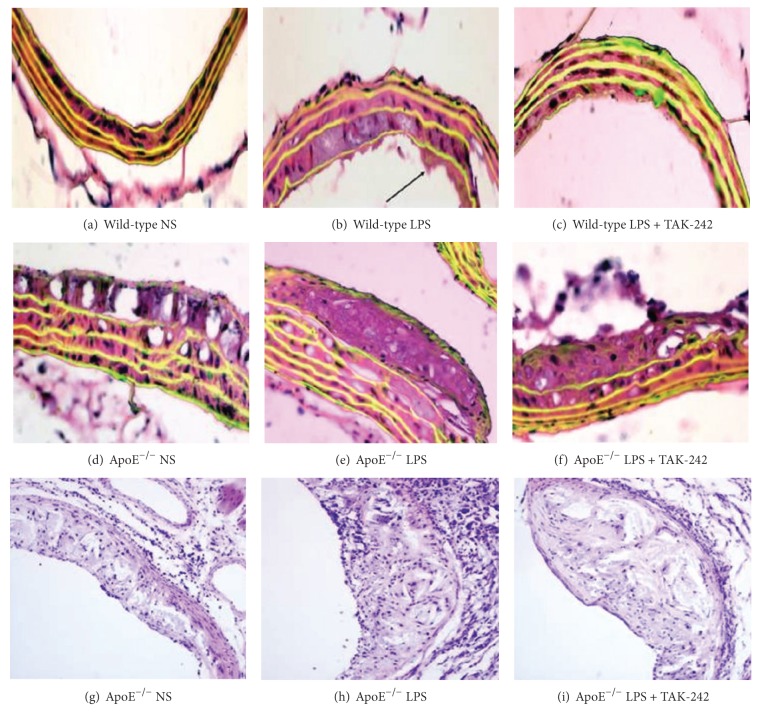
Effects of LPS and TAK-242 on brachiocephalic artery intima-media thickness or aortic sinus plaques. Specimens of HE staining under fluorescence microscopy depicted thickened IMT (arrow) in LPS-treated vessel compared to control artery ((a)–(f), 400x). And HE staining indicated no obvious changes of aortic sinus plaques in ApoE^−/−^ mice ((g)–(i), 100x). NS: physiological saline; LPS: lipopolysaccharide; TAK-242: resatorvid.

Vessel intima-media thickness (IMT) and plaques presence were valid surrogate for assessing preclinical atherosclerosis. To clarify the effects of LPS on atherosclerosis development, IMT in brachiocephalic artery was quantified with fluorescence microscopy after staining with HE.  Figure 3 depicted that in wild-type control mice, the walls of blood vessels were smooth and thin. However, LPS exposure mice were characterized by increased IMT and disarranged endothelium. While in ApoE^−/−^ control mice, fatty streaks and strikingly thickened IMT could be observed. And fibrofatty atherosclerotic plaque with well-defined fibrous caps was present at the initial segment of brachiocephalic artery following LPS intervention in ApoE^−/−^ mice. Ameliorated structural derangements and thinned IMT were noted after LPS and TAK-242 coadministration in both strains of mice.

Additionally, aortic sinus plaques appeared only in ApoE^−/−^ mice, but not in wild-type mice (Table 2). Compared with NS-treated ApoE^−/−^ mice, LPS-primed mice showed a slightly but not significantly larger plaque area. However, increased inflammatory cell infiltration in vascular adventitia and plaques could be observed in ApoE^−/−^ mice after LPS exposure. And TAK-242 could ameliorate evidently atherosclerotic inflammatory response, whereas it showed only a marginal inhibitory effect on plaque area which reached no statistic significance.

### 3.5. Colocalization of TLR4 Expressions in Macrophages from Lung and Aortic Sinus

TLR4 in macrophages is critical in plaque development and pulmonary inflammation. To investigate the implications of macrophage TLR4 in the pathogenesis of plaque and lung injury, colocalized macrophage TLR4 expressions in lung and aortic sinus were performed. Of note, double immunofluorescent staining revealed that TLR4 (green) was mostly enriched in F4/80^+^ (red) macrophages from aortic sinus and lung. The fluorescence intensity of TLR4 and F4/80 double-labeled positive cells increased visibly in the lungs of both mouse strains following LPS injection. And TAK-242 could obviously alleviate these manifestations in lung samples.

**Figure 4:**
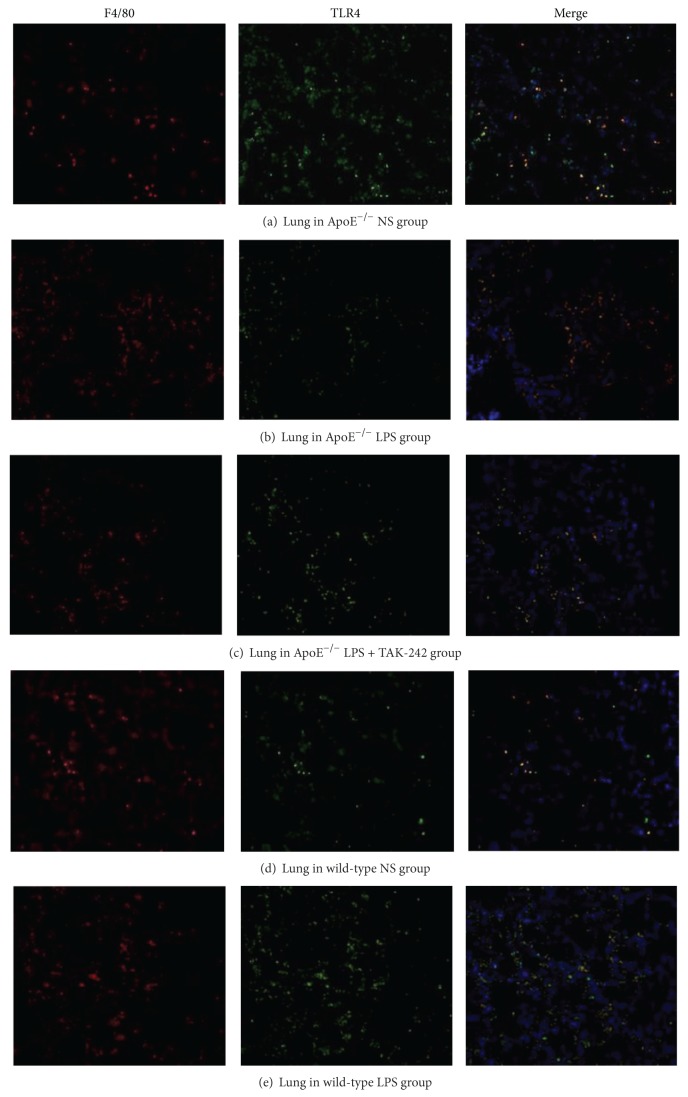

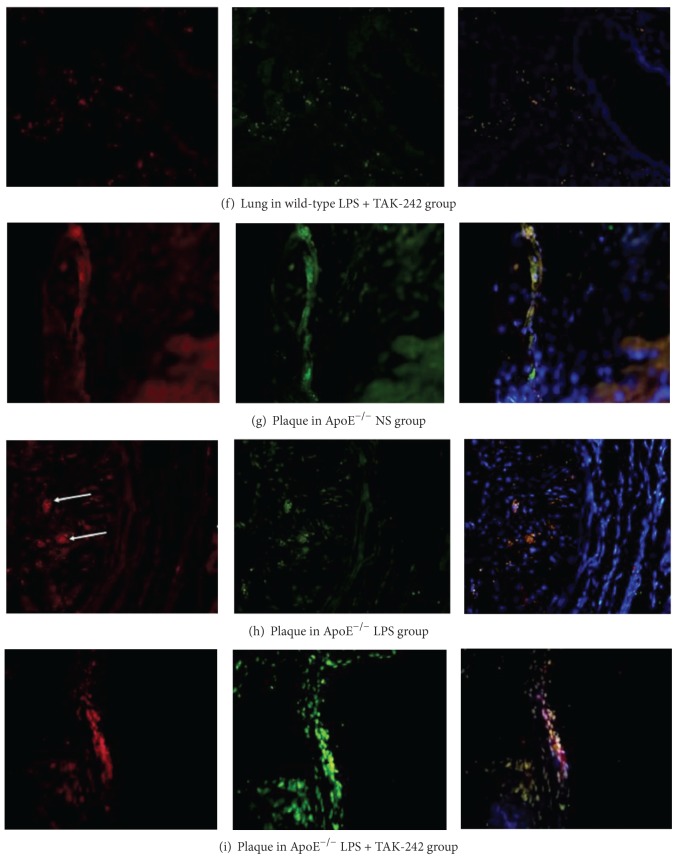
Double immunofluorescence labeling of TLR4 and F4/80^+^ macrophages in lung and aortic sinus plaque. Distribution of TLR4 (green) in macrophages detected by F4/80 (red) from lung ((a)–(f)) and aortic sinus plaques ((g)–(i)). Nuclei were stained with DAPI (blue). The arrow indicated macrophages located at the deeper layers of the plaque after LPS challenge (200x).

When it referred to the effects of LPS on plaques of aortic sinus, TLR4-rich macrophages were evidently increased in the plaques of ApoE^−/−^ mice after LPS treatment. And those macrophages were abundantly expressed in the deeper layers of the plaques. However, TLR4-rich macrophages were reduced and mostly restricted to the subendothelial regions after LPS and TAK-242 coadministration (Figure 4).

### 3.6. Enhanced NF-*κ*B and BAFF Expression in Both Lung and Aortic Sinus after LPS Treatment

**Figure 5:**
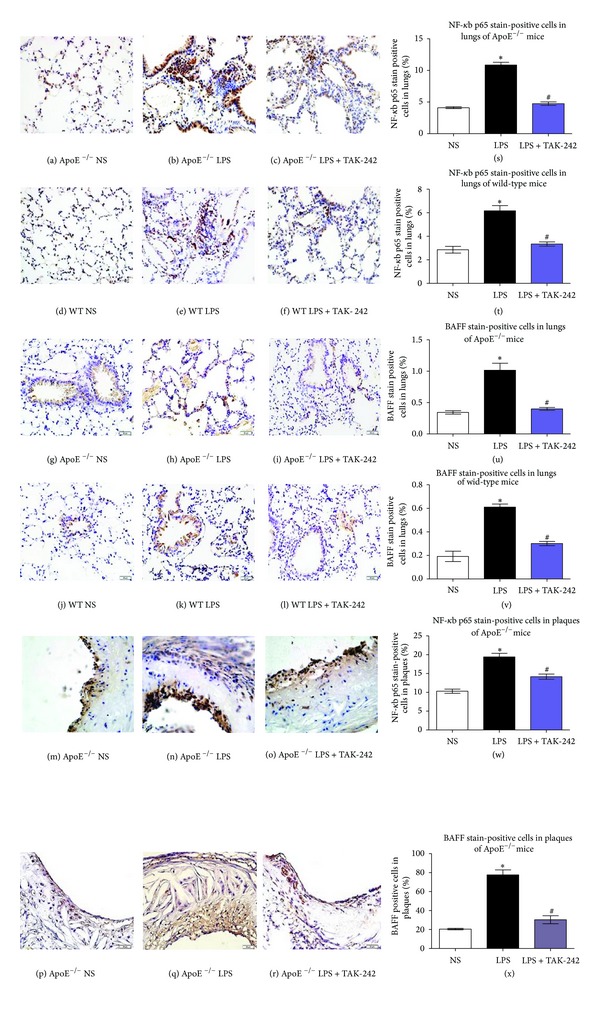
Immunohistochemistry of LPS-exposed ApoE^−/−^ and wild-type mice stained with anti-NF-*κ*B (lung: (a)–(f); aorta: (m)–(o)) and BAFF (lung: (g)–(l); aorta: (p)–(r)). Original magnification: ×400. Histogram ((s)–(x)) represented mean ± SD (*n* = 10). *P < 0.05 versus corresponding NS-exposed mice. ^#^ *P* < 0.05 versus corresponding LPS-exposed mice. WT: wild type; NF-*κ*B: nuclear factor-*κ*B; BAFF: B-cell-activating factor belonging to the TNF family; NS: physiological saline; LPS: lipopolysaccharide; TAK-242: resatorvid.

Macrophage-derived BAFF and NF-*κ*B, initiated by LPS, were essential for autoimmune disease, inflammatory cascade, and atherosclerosis. We focused on whether the changes of NF-*κ*B p65 and BAFF in plaques and lung were synchronized. Interestingly, NF-*κ*B p65 and BAFF expressions were enhanced in lungs after LPS exposure in both mouse strains. Meanwhile, in ApoE^−/−^ mice, the increased expressions of NF-*κ*B p65 and BAFF in plaques were paralleled with what occurred in lung. While these changes were significantly attenuated by TAK-242 plus LPS intervention (Figure 5).

## 4. Discussion 

Our study indicates that LPS induced the production of autoantibody and inflammatory cytokines, accelerated the development of AS, and exacerbated hemorrhagic pulmonary capillaritis in mice fed with high-fat diet. And these manifestations were more obvious in ApoE^−/−^ mice than in wild-type counterparts. While TLR4 antagonist (TAK-242) could strikingly alleviate those pathological changes. Mechanistically, TLR4-mediated activation of NF-*κ*B and BAFF in macrophages may be involved. 

### 4.1. TLR4/NF-*κ*b/BAFF in Hemorrhagic Pulmonary Capillaritis

The mechanism of hemorrhagic pulmonary capillaritis in SLE is still obscure as yet. It is presumed to be immune complex-mediated tissue injury. Barker et al. [3] indicated that the pathogenesis of haemorrhagic pulmonary capillaritis was implicated in B cells as evidenced by reduced alveolar hemorrhage prevalence in Rag1^−/−^ or Igmu^−/−^ mice. This was partly in agreement with our findings. We found that BAFF, a key regulator of B-cell activation and immunoglobulin production, were upregulated in the pathological lungs. Alternatively, apolipoprotein E, which is expressed by lung macrophages, has been shown to modulate lung development in mice, as ApoE^−/−^ mice have diminished developmental alveologenesis, high airways resistance, and more rapid loss of lung recoil with aging [11]. Furthermore, it was well documented [12, 13] that ApoE mimetic peptides were novel treatment approaches for asthma. Similarly, we found that 3 ApoE^−/−^ mice treated with NS developed alveolar hemorrhage. And the role of ApoE lipoprotein in modulating inflammation and immune might be involved [14]. Previous studies reported that NF-*κ*B was activated in SLE patients. Direct transfer of NF-*κ*B p65 into T lymphocytes from SLE patients leads to increased levels of IL-2 promoter activity [15]. Additionally, reports indicated that BAFF enhanced long-term B cell survival primarily through the noncanonical NF-*κ*B pathway, while it promoted immunoglobulin class switching and generation of pathogenic antibodies through the classical pathway [16]. Studies showed that LPS increased BAFF expression by cAMP production in macrophages [17]. The blockage of BAFF prolongs the survival of NZB/W mice and prevents glomerulonephritis and kidney inflammation.

### 4.2. TLR4/NF-*κ*b/BAFF in Atherosclerosis

Simultaneously, we demonstrated that LPS challenge resulted in AS development in both mouse strains. These results were in accordance with previous studies indicating that the aortic walls were thickened by exposure to high-fat diet and repeated LPS injection [18]. Our data were further validated by Vink et al. [19] who claimed vessel adventitial TLR4 activation augmented neointima formation in a periadventitial cuffed mouse model. Unexpectedly, the plaque area showed no statistic significance between LPS or NS treated ApoE^−/−^ mice. The discrepancy may be the fact that the investigation time of 4 weeks was too short to detect the plaque pathological changes. Interestingly, we found that macrophages were mainly located at the deeper layers of the plaque, whereas they were mostly restricted to the subendothelial regions after TAK-242 intervention, which suggested that TAK-242 might play a role in macrophage transmigration. TAK-242 (resatorvid), binding selectively to Cys^747^ in the Toll/interleukin-1 receptor (TIR) domain of TLR4, is a specific inhibitor of TLR4 signaling. There is increasing evidence that TAK-242 improved insulin resistance [20] and endotoxin shock [21]. However, the data on the therapeutic effects of TAK-242 on atherosclerosis were not available at present.

Additionally, we found that BAFF expressions in aortic plaques were enhanced in LPS-challenged ApoE^−/−^ mice. In support of this finding, Kyaw et al. [22] demonstrated that in ApoE^−/−^ mice, anti-BAFFR (BAFF-receptor) antibody treatment ameliorated atherosclerosis progression.

### 4.3. Autoimmune Response and LPS-TLR4

A previous investigation reported that TLR4 upregulation at protein or gene level is sufficient to break immunologic tolerance. In Fas^lpr/lpr^ mutation mice, TLR4 deficiency markedly decreased autoantibody production of anti-dsDNA and anti-ribonucleoprotein (RNP) and improved glomerulonephritis. This was somewhat in agreement with our previous findings that ANA and anti-dsDNA were increased in LPS-challenged ApoE knockout and wild-type mice [10]. Surprisingly, TAK-242 could significantly reduce the anti-dsDNA levels rather than ANA, which suggested the complexities of autoantibody production.

In particular, a large body of literature has been published that TLR4 expression was enhanced in atherosclerotic plaques. And our results at present confirmed those findings. While in our previous work, we found decreased TLR4 expression and even lower TLR4 levels by LPS challenge in spleen specimens of ApoE^−/−^ mice [10]. The plausible explanations for this seemingly contradiction were as follows: spleen theoretically is the largest peripheral immune organ in vivo and it plays an important role in initiating immune responses. While lung and artery are immune-targeted effector organ. So immune responses to microbial challenge in various tissues may not be the same, and TLRs in different tissue environments may present heterogeneity. And our opinion was exemplified by Davies et al. who claimed differential regulation of toll-like receptor signalling in spleen and Peyer's patch dendritic cells [23] as well as by Veeresh et al. who claimed tissues-specific expression pattern of swine TLRs 1-10 [24].

### 4.4. Inflammatory Mediators (IFN-*γ*, TNF-*α* and IL-1*β*)

Our results showed a significant increase of TNF-*α*, IFN-*γ*, and IL-1*β* in C57BL/6 mice after LPS stimulation, to a larger extent in ApoE^−/−^ mice. It has been reported that cytokines of TNF-*α*, IFN-*γ*, and IL-1, -6, -10, -15, and IL-18 are upregulated in SLE patients and play important roles in the inflammatory processes that cause organ damages [25, 26]. Alternatively, these results corroborated that the findings the production of T helper-1 cytokines (IL-12, and IFN-*γ*) were exaggerated in ApoE-deficient mice [27].

In conclusion, our findings suggested TLR4/NF-*κ*b/BAFF in macrophages may be a possible common autoimmune pathway that resulted in multisystem involvement of lung injury and AS in SLE. The exacerbation of hemorrhagic pulmonary dapillaritis and as induced by LPS was more serious in ApoE^−/−^ than in wild-type C57BL/6 mice. Based on these findings, TLR4 signal could be a therapeutic target in AS and lung inflammation injury. However, the animal model is not fully representative of the human disease, and therefore the results observed must be interpreted with caution. Additionally, due to the complexity in the regulation of autoimmunity in SLE, the roles of other TLRs in our model thus warrant further examination.

## Figures and Tables

**Table 1:** Sera ANA (ug/mL) and anti-dsDNA (U/mL) levels in LPS-challenged mice.

	ApoE^−/−^ (*n* = 10)		Wild-type mice (*n* = 10)	
	ANA	Anti-dsDNA	ANA	Anti-dsDNA
NS	93.4 ± 7.9^$^	(2.7 ± 0.5) × 10^4$^	40.8 ± 5.2	(2.4 ± 0.6) × 10^4^
LPS	145.5 ± 19.2^∗$^	(8.0 ± 1.3) × 10^4∗$^	58.5 ± 8.2*	(4.1 ± 0.4) × 10^4∗^
LPS + TAK-242	125.6 ± 21.3^$^	(4.2 ± 0.8) × 10^4#$^	44.8 ± 5.9	(2.9 ± 0.9) × 10^4#^

*P < 0.05  versus  corresponding NS group, ^#^P < 0.05  versus  corresponding LPS group, and ^$^P < 0.05  versus  wild-type mice. ANA: anti-nuclear antibody (ANA); anti-dsDNA: anti-double-stranded deoxyribonucleic acid; NS: physiological saline; LPS: lipopolysaccharide; TAK-242: resatorvid.

**Table 2:** Intima-media thickness of brachiocephalic artery and plaque area of aortic sinus in ApoE^−/−^ and wild-type mice.

	ApoE^−/−^ (*n* = 10)		Wild-type mice (*n* = 10)	
	IMT (*μ*m)	Plaque area (%)	IMT (*μ*m)	Plaque area (%)
NS	42.1 ± 1.9^$^	20.2 ± 2.3	20.5 ± 3.6	Not arise
LPS	75.8 ± 5.8^∗$^	22.1 ± 4.6	39.7 ± 7.4*	Not arise
LPS + TAK-242	55.4 ± 6.4^$#^	20.7 ± 3.2	27.3 ± 4.1^#^	Not arise

*P < 0.05  versus  corresponding NS group, ^#^P < 0.05  versus  corresponding LPS group, and ^$^P < 0.05  versus  wild-type mice. IMT: intima-media thickness; NS: physiological saline; LPS: lipopolysaccharide; TAK-242: resatorvid.
